# Molecular Dynamics Simulation of Improving the Physical Properties of Polytetrafluoroethylene Cable Insulation Materials by Boron Nitride Nanoparticle under Moisture-Temperature-Electric Fields Conditions

**DOI:** 10.3390/polym11060971

**Published:** 2019-06-03

**Authors:** Xu Hua, Li Wang, Shanshui Yang

**Affiliations:** Department of Electrical Engineering, Nanjing University of Aeronautics and Astronautics, Nanjing 210016, China; huaxu@nuaa.edu.cn (X.H.); yshanshui@nuaa.edu.cn (S.Y.)

**Keywords:** glass transition temperature, mechanical properties, thermal conductivity, relative dielectric constant

## Abstract

The physical properties in amorphous regions are important for the insulation aging assessment of polytetrafluoroethylene (PTFE) cable insulation materials. In order to study the effect of boron nitride (BN) nanoparticles on the physical properties of PTFE materials under moisture, temperature, and electric fields conditions at the molecular level, the amorphous region models of PTFE, BN/PTFE, water/PTFE, and water/BN/PTFE were respectively constructed by molecular dynamics (MD) simulation. The mechanical properties including Young’s modulus, Poisson’s ratio, bulk modulus, and shear modulus, along with glass transition temperature, thermal conductivity, relative dielectric constant, and breakdown strength of the four models have been simulated and calculated. The results show that the mechanical properties and the glass transition temperature of PTFE are reduced by the injection of water molecules, whereas the same, along with the thermal conductivity, are improved by incorporating BN nanoparticles. Moreover, thermal conductivity is further improved by the surface grafting of BN nanoparticles. With the increase of temperature, the mechanical properties and the breakdown strength of PTFE decrease gradually, whereas the thermal conductivity increases linearly. The injection of water molecules increases the water content in the PTFE materials, which causes a gradual increase in its relative dielectric constant. This work has shown that this effect is significantly reduced by incorporation of BN nanoparticles. The variation of physical properties for PTFE and its composites under the action of moisture, temperature, and electric fields is of great significance to the study of wet, thermal, and electrical aging tests as well as the life prediction of PTFE cable insulation materials.

## 1. Introduction

Polytetrafluoroethylene (PTFE) insulation materials have excellent thermodynamic properties in addition to stable chemical and dielectric properties. These materials also have special surface properties, a low friction coefficient, ablation resistance, and self-lubrication [1,2,3]. PTFE, in comparison with low-density polyethylene (LDPE), is more difficult and expensive to process, but its continuous operating temperature reaches 533 K, which makes it more suitable for high temperature applications. The glass transition temperature (*T*g) of PTFE is also about 145 K higher than that of LDPE [4]. In addition, PTFE has a low dissipation factor, good stability over a wide frequency range, high volume resistivity, and high breakdown strength [5]. Therefore, PTFE is widely used in special fields such as aviation cables, high voltage switch breakers, sealing materials, insulator materials, and printed circuit board materials [6,7]. In particular in the aviation electrical system, transmission cables are mostly insulated by PTFE materials. During the flight of aircraft, aviation cables have been affected by high altitude radiation, high temperature, electricity, vibration, moisture, and other factors for a long time [8,9,10]. Therefore, higher requirements have been put forward for the physical and chemical properties of PTFE materials. With the development of nanotechnology, nanocomposites have been widely used in the field of insulation dielectrics [11]. Polymer nanocomposites bring about significant improvements in various properties. Surface functionalization of nanoparticles is capable of increasing the characteristic breakdown strength [12,13]. The composites with low filling concentration allow charges to leak out of the material slowly to avoid space charge accumulation [14,15]. The incorporation of highly thermal conductive nanoparticles can effectively improve the thermal conductivity of polymers, and surface modification of nanoparticles can further improve thermal conductivity [16]. However, the thermal conductivity of composites has been related to many factors such as filler morphology, concentration, and inherent thermal conductivity as well as the strength of bonding between polymer and filler [17,18,19]. Good interfacial adhesion between the polymer matrix and nanoparticles can significantly improve long-term mechanical behaviors of nanocomposite such as wear and abrasion resistance, fatigue, and creep [20]. Boron nitride (BN) nanoparticles have a low dielectric constant of 4 (1 GHz) and a low loss factor of 0.00035 (1 GHz), which makes them very suitable for high frequency applications and polymer composites. BN filler has high intrinsic thermal conductivity and low thermal expansion coefficient, which can effectively improve the thermal conductivity of polymer-based materials, reduce the thermal expansion coefficient, and slightly reduce the loss factor [21]. Therefore, the physical properties of PTFE enhanced by BN nanoparticles under different conditions are studied in this paper.

At present, the aging evaluation of aeronautical cable insulation materials mainly focus on the statistics of cable operation data and the research of accelerated aging experiments. However, there are still some deficiencies in the research of aging and modification mechanisms of insulating materials from the microscopic point of view, especially for BN nanoparticle-modified PTFE materials; there are few reports on the change of their physical properties under the synergistic effect of water, temperature, and electric fields. With the rapid development of computer technology and the continuous updating of high-performance servers, molecular simulation technology for simulating the changes of molecular structure and properties of materials has been widely used [22,23]. Scholars all over the world have used molecular simulation technology to study the cracking of insulating paper in transformers [24], the damage mechanism of water and acid on insulating oils [25], the effect of aging gas products on thermal stability of polyethylene [26], and the thermodynamic properties of epoxy resin/silica composites [27]. Molecular simulation can not only accurately calculate the physical properties of materials but also illustrate the phenomena that macroscopic experiments fail to examine.

The purpose of this paper is to understand the change in the physical properties of PTFE insulating materials modified by BN nanoparticles under moisture-temperature-electric field conditions by molecular dynamics (MD) simulation. It can provide guidance for the performance degradation of PTFE cable materials during thermal, wet, and electrical aging, which would avoid using the aging test that has high cost and long periods of testing. At the same time, Young’s modulus, Poisson’s ratio, thermal conductivity, and the relative dielectric constant of PTFE under different conditions can be used as modeling parameters for the aging model of PTFE cables. In addition, the mechanical properties, thermal conductivities, relative dielectric constants, and breakdown strengths of boron nitride-incorporated PTFE with and without water offered by this study could serve as good references for the field of polymer coatings.

## 2. Modeling and Simulation Details

### 2.1. Model Building

The insulating materials of PTFE cable consists of the crystalline region and the amorphous region. The molecular arrangement of the crystalline region is regular, but the molecular arrangement of the amorphous region is relaxed. The insulation deterioration mostly begins in the amorphous region of the insulating layer. Therefore, the amorphous region model of PTFE materials was constructed to explain the effect of water molecules and BN nanoparticles on its physical properties. The molecular models of PTFE, water/PTFE, BN/PTFE, and water/BN/PTFE were built by LAMMPS software and visualized by OVITO software. The details of the modeling are as follows: Firstly, a long PTFE chain with a degree of polymerization of 200 was constructed and its structure was optimized with the COMPASS force field [28]. This force field can accurately predict the structural, conformation, and microscopic properties of a broad range of organic and inorganic molecules. The total energy of PTFE optimized is −5243 kcal/mol, CF_3_-CF_2_ bond length is 1.53 Å, and C-F bond length is 1.36 Å; the structure factors are in good agreement with Ref. [29]. Then, the amorphous region model of PTFE materials was further constructed; the region contains four optimized PTFE molecular chains. Relevant references show that when the degree of polymerization of polymer molecular chains reaches 10, the molecular cohesive energy density reaches a relatively stable value, and the structural parameters are consistent with the experimental values [30,31,32]. Therefore, it is reasonable to choose a molecular chain with a degree of polymerization of 200 as a basic unit to construct the amorphous region model of PTFE materials.

**Figure 1:**
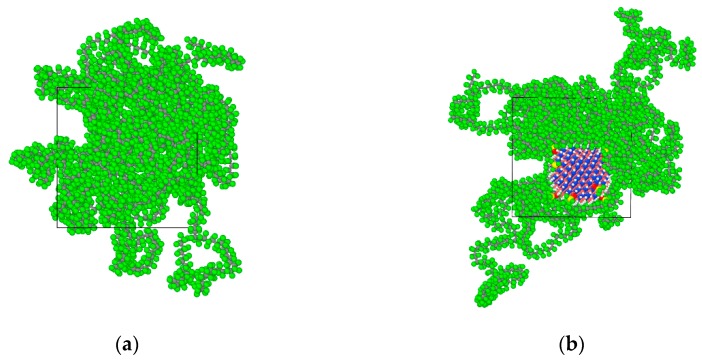

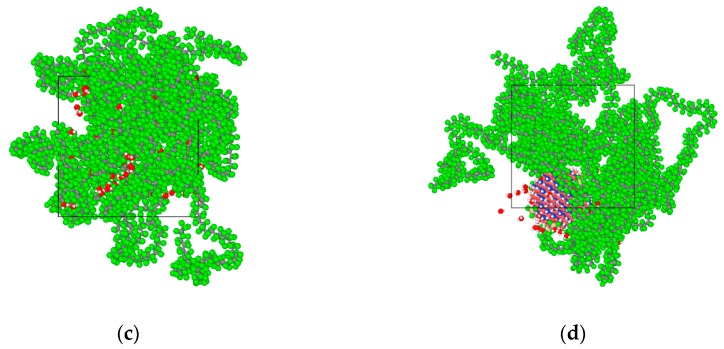
Polytetrafluoroethylene (PTFE) model and its composite models: (**a**) PTFE model; (**b**) boron nitride (BN)/PTFE model; (**c**) water/PTFE model; and (**d**) water/BN/PTFE model.

The initial density of the amorphous region was set at 2.2 g/cm^3^. The Ewald summation method was used to calculate the electrostatic interaction. The Atom Based method was used to calculate the van der Waals interactions. After 3000 iterations of structural optimization, a three-dimensional amorphous region model with stable structure was obtained. Its cell size is 39.26 Å × 39.26 Å × 39.26 Å. The corresponding PTFE model is shown in Figure 1a. Ten tetrafluorosilanes were grafted onto the surface of BN nanoparticles with a radius of 1 nm and then introduced into the pure PTFE model to obtain the BN/PTFE composite model as shown in Figure 1b. The water/PTFE composite model and water/BN/PTFE composite model were obtained by incorporating water molecules with 2% mass fraction into the PTFE and BN/PTFE composite models as shown in Figure 1c,d. The initial conformation of PTFE composite models for MD simulation was obtained by the same structural optimization process. Because the COMPASS force field cannot calculate the N particle, the structure of BN nanoparticle was optimized by Universal force field [33]. In order to reduce the contrast error, all the following MD simulations were carried out with the Universal force field.

### 2.2. Model Optimizing and Annealing Details

The details of the MD simulation are as follows: In order to make the initial conformation of PTFE and its composite models close to that of the molecular space structure in real materials, the initial model was optimized by energy minimization and geometry optimization. The smart method was used to adjust the bond length and bond angle between atoms, and the maximum number of iterations was set to 10,000 times to ensure the successful completion of optimization. In order to remove residual stress and make the distribution of holes more reasonable, the corresponding initial model was annealed five times. In each cycle, the MD simulation temperature decreased from 800 K to 100 K under the NVT ensemble (constant number of particles, volume and temperature), and then increased from 100 K to 800 K. The time of each cycle was set to 1000 ps, and the temperature rise and fall intervals were set at 50 K. This process reduces the irrationality of the local structure, which provides a reasonable equilibrium conformation for the calculation of physical properties of the PTFE model and its composite models.

## 3. Simulation Methods and Results

### 3.1. Glass Transition Temperature

**Figure 2:**
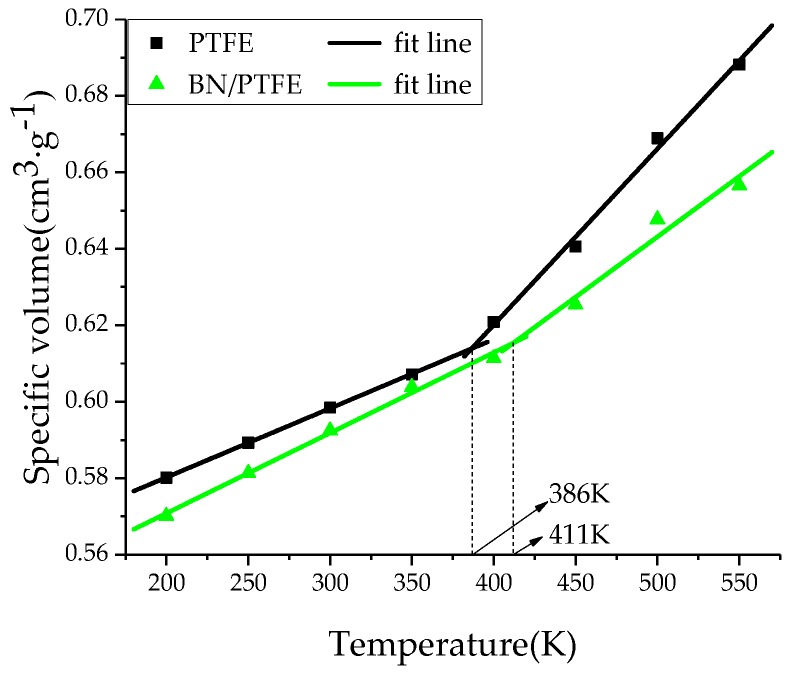
Determination of *T*g for PTFE and BN/PTFE models.

**Figure 3:**
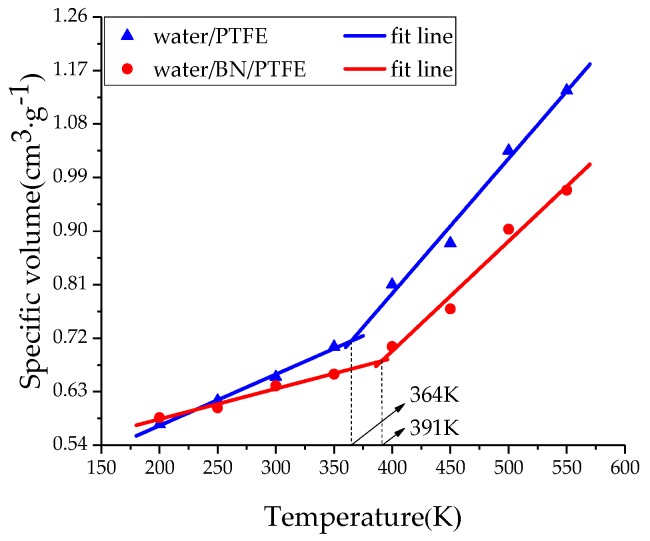
Determination of *T*g for water/PTFE and water/BN/PTFE models.

The amorphous region of PTFE insulating materials is in a glassy state at room temperature. When *T*g is reached, PTFE materials are transformed into a rubbery state. This transformation process will lead to significant changes in various properties of PTFE materials, such as free volume, mechanical properties, and the stability against degradation. Therefore, the determination of *T*g is of great significance for studying the thermodynamic properties of insulating materials. By heating up to 550 K and then cooling down to 200 K with a rate of 50 K/200 ps, the system at equilibrium experienced an annealing process. Under atmospheric pressure (1 atm), a simulation under the NPT ensemble with the Andersen thermostat method was performed. The specific volume-temperature method [34] was used to determine the *T*g of PTFE and its composite models. In Figure 2, the *T*g of PTFE model is 386 K and that of BN/PTFE model is 411, which is 25 K higher than that of the PTFE model. It shows that the incorporation of BN nanoparticles improves the stability against degradation of the PTFE model. Figure 3 shows that the *T*g of the water/PTFE model is 364 K, which is 22 K lower than that of the PTFE model; the *T*g of the water/BN/PTFE model is 391 K, which is 20 K lower than that of the BN/PTFE model; and this indicates that the incorporation of water molecules reduces the *T*g of both the PTFE model and the BN/PTFE model. The *T*g of the water/BN/PTFE model is 27 K higher than that of water/PTFE model, which indicates that BN nanoparticles reduce the effect of water on the thermal property of PTFE materials. The corresponding macroscopic phenomenon is that the heat resistance of PTFE insulating materials can be obviously improved by adding BN nanoparticles, but after wet aging, the thermal property of PTFE insulating materials begins to decrease, so BN nanoparticles can improve the *T*g of PTFE before and after wet aging to a certain extent.

### 3.2. Mechanical Properties

The static constant strain method [35,36] was used to calculate the mechanical parameters of the PTFE model, the BN/PTFE model, the water/PTFE model, and the water/BN/PTFE model. The strain range was set within 0.03. The strain was applied along the six directions of *xx*, *yy*, *zz*, *yz*, *zx*, and *xy* of the PTFE model. Then, the corresponding stress was obtained. According to these calculated results, the stiffness coefficient *C*_ij_ can also be obtained. The elastic stiffness matrix shows that the PTFE materials is anisotropic, but the values of *C*_14_, *C*_15_, *C*_16_, *C*_24_, *C*_25_, *C*_26_, *C*_34_, *C*_35_, *C*_36_, *C*_45_, *C*_46_, and *C*_56_ are all approximate to zero, so the model can be regarded as an isotropic material for simplified calculation.

(1) $$\left[\begin{array}{c} \delta_{1}\\ \delta_{2}\\ \delta_{3}\\ \delta_{4}\\ \delta_{5}\\ \delta_{6} \end{array}\right]=\left[\begin{array}{cccccc} C_{11} & C_{12} & C_{13} & C_{14} & C_{15} & C_{16}\\ C_{21} & C_{22} & C_{23} & C_{24} & C_{25} & C_{26}\\ C_{31} & C_{32} & C_{33} & C_{34} & C_{35} & C_{36}\\ C_{41} & C_{42} & C_{43} & C_{44} & C_{45} & C_{46}\\ C_{51} & C_{52} & C_{53} & C_{54} & C_{55} & C_{56}\\ C_{61} & C_{62} & C_{63} & C_{64} & C_{65} & C_{66} \end{array}\right]•\left[\begin{array}{c} \varepsilon_{1}\\ \varepsilon_{2}\\ \varepsilon_{3}\\ \varepsilon_{4}\\ \varepsilon_{5}\\ \varepsilon_{6} \end{array}\right]$$

The Lame coefficients *λ* and *μ* are calculated from the calculated stiffness coefficients as follows:

(2) $$\lambda =\frac{1}{3}(C_{11}+C_{22}+C_{33})-\frac{2}{3}(C_{44}+C_{55}+C_{66})$$

(3) $$\mu =\frac{1}{3}(C_{44}+C_{55}+C_{66})$$

The specific mechanical parameters can be obtained by the Lame coefficient as follows:

(4) $$E=\mu \frac{3\lambda +2\mu }{\lambda +\mu }$$

(5) $$v=\frac{1}{2}\frac{\lambda }{\lambda +\mu }$$

(6) $$B=\lambda +\frac{2}{3}\mu$$

(7) G=μ

**Figure 4:**
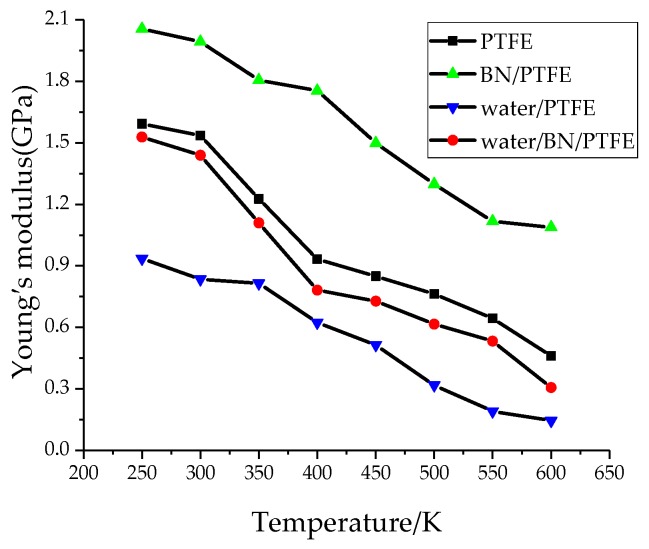
Young’s modulus versus temperature.

**Figure 5:**
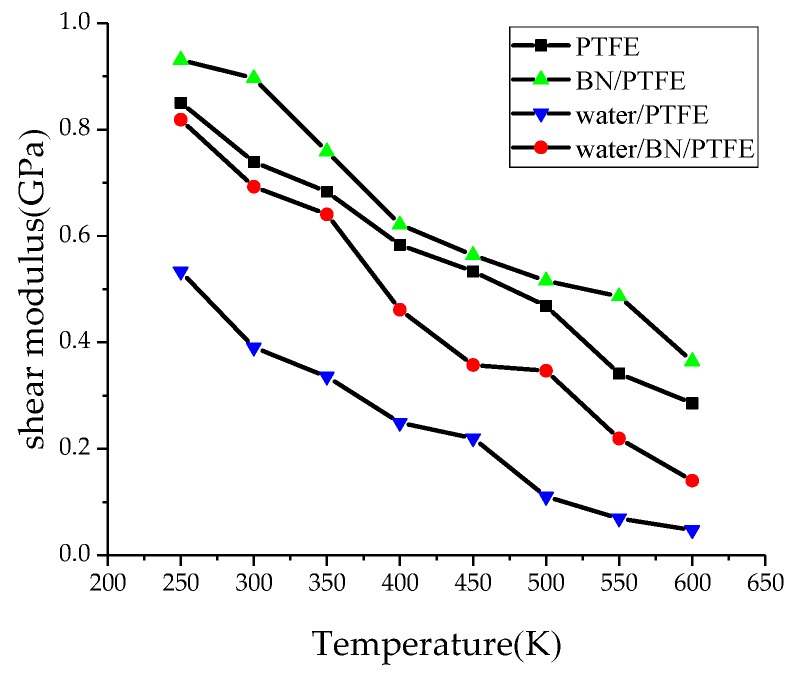
Shear modulus versus temperature.

**Figure 6:**
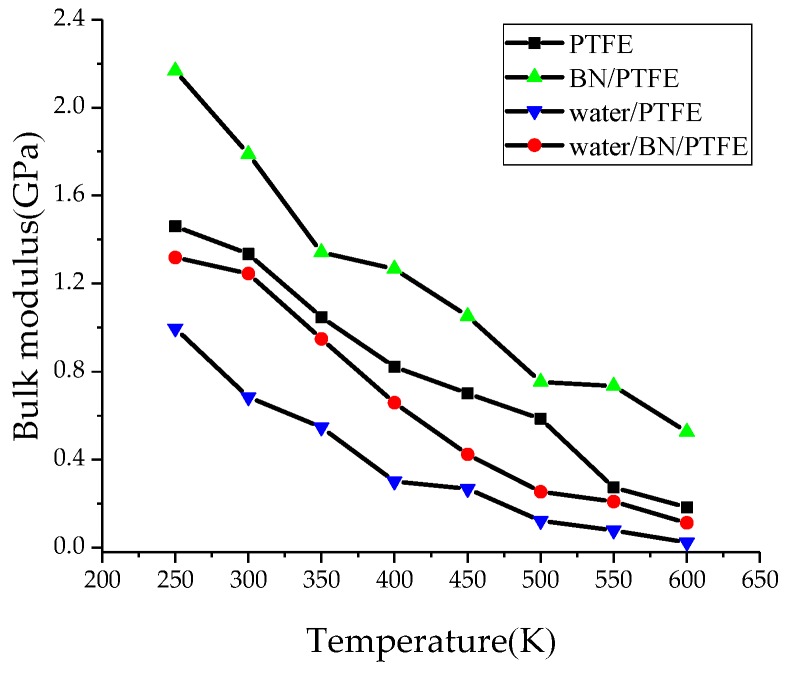
Bulk modulus versus temperature.

**Figure 7:**
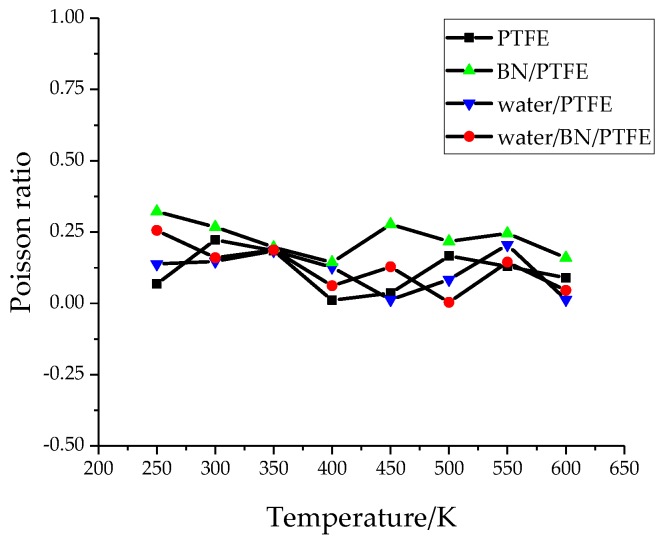
Poisson’s ratio versus temperature.

Young’s modulus *E* is used to characterize material rigidity, Poisson’s ratio *v* is used to characterize material plasticity, bulk modulus *B* is used to characterize material incompressibility, and shear modulus *G* is used to characterize material’s ability to resist shear strain. Ref. 21 shows that Young’s modulus increases with increasing BN filler content, and the highest Young’s modulus is 3 GPa when the content of BN is 25%. The Young’s modulus of PTFE is 1.6 GPa and that of BN/PTFE is 2.1 GPa, as calculated by MD simulation. Figure 4, Figure 5, Figure 6 and Figure 7 are the curves of Young’s modulus, shear modulus, bulk modulus, and Poisson’s ratio with varying temperatures, respectively. These figures show that the Young’s modulus, shear modulus, and bulk modulus of the four models decrease gradually with the increase of temperature. Young’s modulus, shear modulus, and bulk modulus of the BN/PTFE model are always at the top of the four curves in each figure, while those properties of water/PTFE model are always at the bottom of the four curves, which indicates that the mechanical properties of the PTFE materials doped with BN nanoparticles have been improved to a certain extent. After wet aging of PTFE materials, the injection of water molecules reduces the mechanical properties of PTFE materials by a large margin. Meanwhile, the mechanical properties of water/BN/PTFE model at various temperatures are larger than those of the water/PTFE model, which indicates that BN nanoparticles have a certain inhibitory effect on the reduction of mechanical properties of PTFE model after wet aging. Poisson’s ratio of the four models decreases slightly with the increase of temperature, and Poisson’s ratio of the BN/PTFE model is always higher than that of the other three models. It shows that temperature has little effect on Poisson’s ratio, and BN nanoparticles can slightly increase the Poisson’s ratio of the PTFE model.

### 3.3. Thermal Conductivity

**Figure 8:**
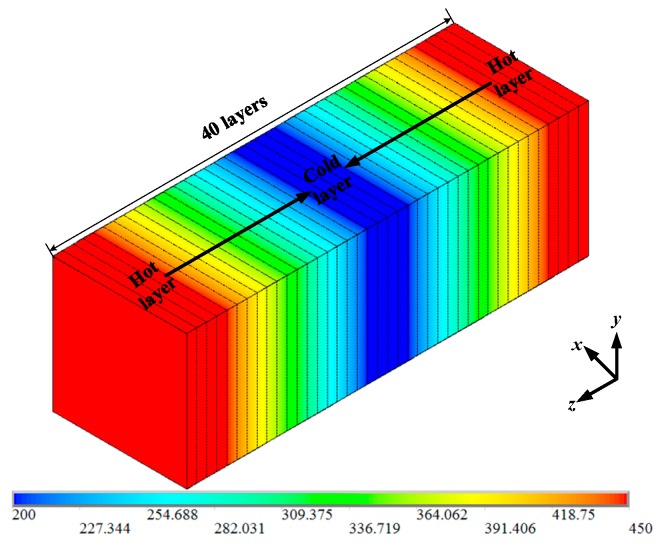
Temperature distribution between cold and hot layers.

**Figure 9:**
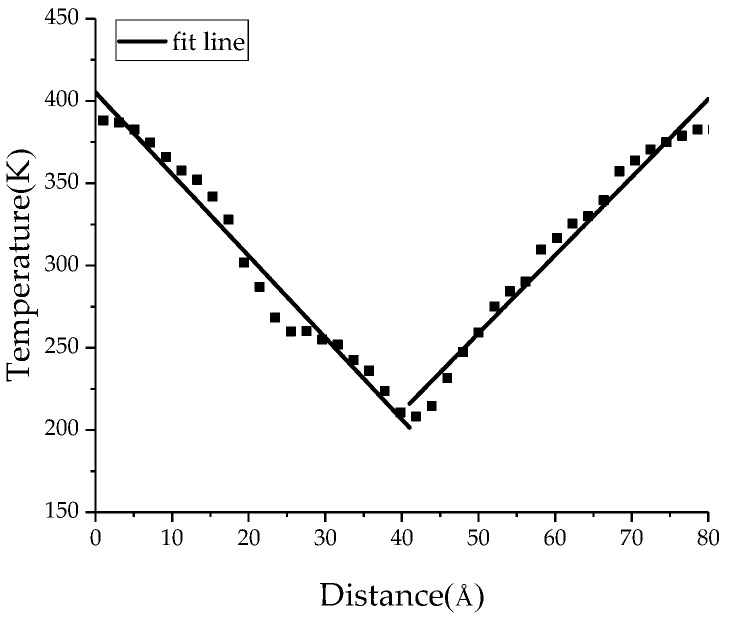
Temperature variation along *z*-axis distance.

In order to study the influence of BN nanoparticles on the thermal conductivity of PTFE insulating materials, the thermal conductivity of the PTFE model, the BN/PTFE model of tetrafluorosilane grafting, and the BN/PTFE model of non-grafting at different temperatures were calculated by the reverse non-equilibrium molecular dynamics (RNEMD) method [37,38,39]. The specific steps for this are as follows: Firstly, the PTFE model was divided into 40 layers along the *z*-axis direction, which was defined as having a hot layer at both ends and a cold layer in the middle; secondly, the atoms with the lowest kinetic energy in the hot layer will exchange energy periodically with those with the highest kinetic energy in the cold layer, and the energy exchange process is shown in Figure 8; finally, the exchange step was set to 0.1 ps, and the exchange frequency is 1000. After completing the primary energy exchange, the energy flux between the cold layer and the hot layer will produce a temperature gradient, and the primary thermal conductivity calculation will be completed. The temperature variation between the cold layer and the hot layer is shown in Figure 9. The temperature gradient d*T*/d*z* was obtained by linear fitting method. The energy flux was calculated by Equation (8), *A* is the sectional area perpendicular to the direction of energy flux, and Δ*E* is the energy flowing in and out. The thermal conductivity was calculated by Equation (9); *J* is the energy flux along *z* axis, and *T* is the temperature. The energy flux is opposite to the direction of temperature gradient.

(8) $$\left|J\right|=\frac{1}{2A}\frac{\Delta E}{\Delta t}$$

(9) $$\lambda =-\frac{J}{dT/dz}$$

**Figure 10:**
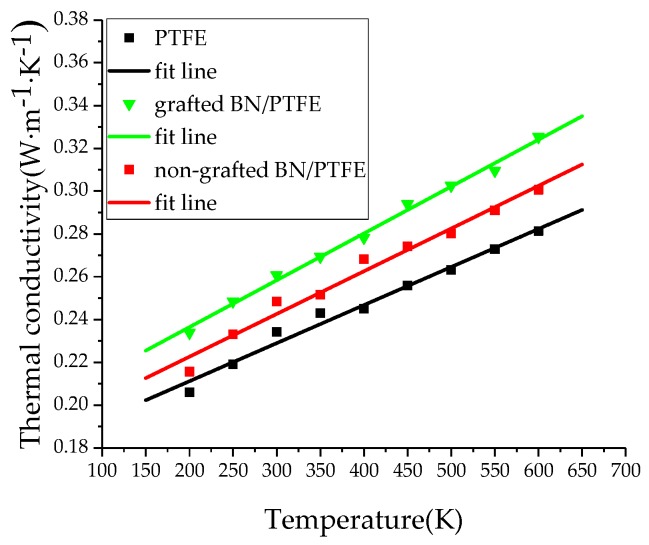
Thermal conductivity versus temperature for three models.

The thermal conductivity of the PTFE model, grafted BN/PTFE model, and non-grafted BN/PTFE model at different temperatures was calculated based on the MD simulation process. The thermal conductivity of the PTFE model at 300 K was 0.234 W·M^−1^·K^−1^, which was close to the experimental value of 0.25 W·M^−1^·K^−1^ [40]. The thermal conductivity of the grafted BN/PTFE model at 300 K was 0.261 W·M^−1^·K^−1^. Figure 10 is a scatter plot of thermal conductivity for three models, varying with temperature. It shows that the thermal conductivity of the PTFE model, grafted BN/PTFE model, and non-grafted BN/PTFE model increases near linearly with temperature, and the thermal conductivity relationship of the three models at different temperatures is as follows: grafted BN/PTFE model > non-grafted BN/PTFE model > PTFE model. It shows that doping BN nanoparticles can improve the thermal conductivity of PTFE materials. Moreover, the thermal conductivity of PTFE materials was further improved by using tetrafluorosilanes to graft the surface of BN nanoparticles with a grafting ratio of 10%. The combining ability of BN nanoparticles with PTFE materials is enhanced after grafting modification, the dispersion degree of BN nanoparticles in PTFE materials is improved, and the corresponding thermal conductivity is further improved.

### 3.4. Dielectric Properties

#### 3.4.1. Relative Dielectric Constant

Under a certain temperature, the relative dielectric constant is calculated by using the fluctuation of the total dipole moment. The theoretical equation is shown in Equation (10) [41,42]. In order to study the dielectric properties of PTFE composite models with 1% water, the dynamic equilibrium of 100 ps under a NVT ensemble was performed for the PTFE model, 1% water/PTFE model, BN/PTFE model, and 1% water/BN/PTFE model. Further simulation ran under the NPT ensemble for 100 ps. The temperature was kept at 300 K and pressure maintained at 101 kPa. The dipole moment in the NPT ensemble was calculated and collected, and the relative dielectric constant of the corresponding model was calculated according to Equation (10).

(10) $$\varepsilon =1+\frac{\langle M^{2}\rangle -\langle M\rangle^{2}}{3\langle V\rangle k_{B}T\varepsilon_{0}}$$

*M* is the total dipole moment of each frame’s dynamic conformation, *k_B_* is the Boltzmann constant, *V* is the volume of the amorphous region, *T* is the temperature, and *ε*_0_ is the vacuum dielectric constant.

**Figure 11:**
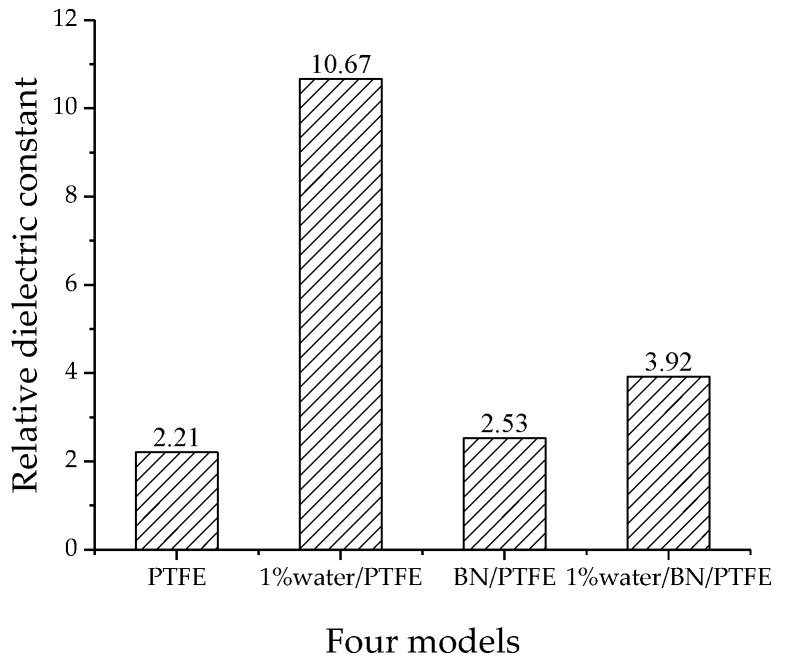
Relative dielectric constant of four models.

The relative dielectric constants of the four models are shown in Figure 11. The relative dielectric constant of the PTFE model is 2.21, the 1% water/PTFE model is 10.67, the BN/PTFE model is 2.53, and the 1% water/BN/PTFE model is 3.92. The experimental relative dielectric constant of PTFE insulating materials is generally about 2.1~2.2, while that of BN-added composites is about 2.2~3.25 [22]. Therefore, the relative dielectric constant calculated by this method has a high accuracy for the PTFE system.

The BN/PTFE composites have good moisture resistance, and the maximum moisture absorption rate is only 0.11% [43] while the moisture absorption requirement of intermetallic dielectrics is less than 1% [44]. Therefore, in order to study the effect of water content on the dielectric properties of PTFE dielectric materials, the PTFE model and BN/PTFE model were mixed with 0~1% water, and the water content increment step was 0.1%. The technical details of the water-cut model are as follows: Firstly, the number of water molecules *n* was calculated according to the mass fraction of water molecules and the total mass of four PTFEs, as shown in Equation (11); secondly, the free volume in the PTFE model was calculated according to the total volume of the amorphous region and the occupied volume of PTFE molecules, as shown in Equation (12); finally, the density of water molecules in free volume was calculated according to the mass of water molecules and the free volume of PTFE model, as shown in Equation (13), and then the PTFE water-cut model corresponding to different water content (0~0.1%) was established.

(11) $$\frac{18n}{18n+M_{\mathrm{PTFE}}}=W_{\mathrm{water}}$$

(12) $$V_{\mathrm{free}}=V_{\mathrm{total}}-V_{\mathrm{occupied}}$$

(13) $$\rho_{\mathrm{water}}=\frac{18n}{N_{A}\times V_{\mathrm{free}}}$$

*W*_water_ is the mass fraction of water, *M*_PTFE_ is the total mass of the PTFE molecule, *n* is the number of water molecules, *V*_free_ is the total volume, *V*_total_ is the total volume, *V*_occupied_ is the occupied volume of PTFE, *N_A_* is the Avogadro constant, and *ρ*_water_ is the density of water molecule in the free volume.

**Figure 12:**
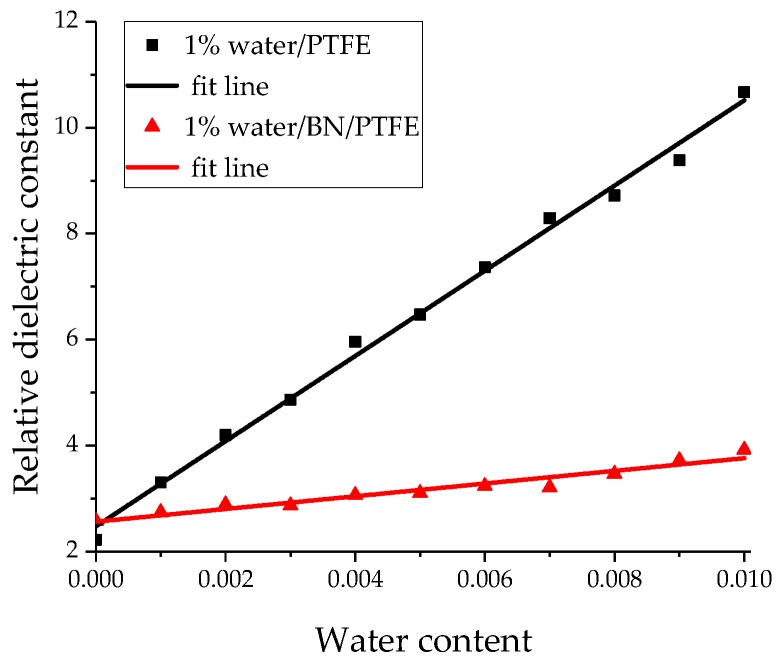
Variation of relative dielectric constant with water content.

The relative dielectric constants of different water content models were obtained by the above dynamic simulation process and the equation of dielectric constant calculation. Figure 12 shows the curve of relative dielectric constant of the water/PTFE model and the water/BN/PTFE model varying with water content. The relative dielectric constant of the two models increases with water content, and the slope of the relative dielectric constant for PTFE/BN water-cut model is lower than that of PTFE water-cut model, which indicates that the dielectric constant of the PTFE water-cut model decreases when BN nanoparticles are incorporated. This is due to the strong charge transfer and adsorption properties of BN nanoparticles, which reduces the dipole moment fluctuation of the PTFE water-cut model in the process of MD simulation. Therefore, the relative dielectric constant of PTFE modified by BN nanoparticles changes less after wet aging.

**Figure 13:**
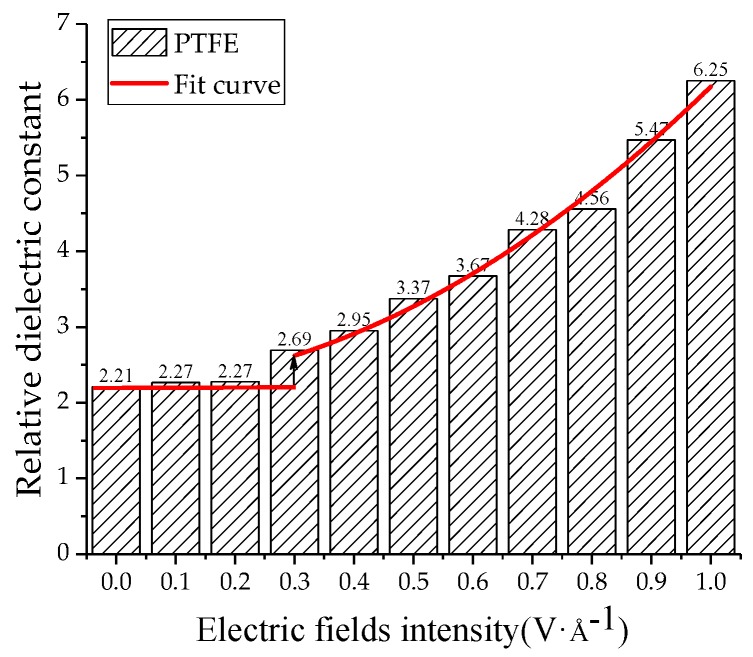
Variation of relative dielectric constant of PTFE model with electric fields.

**Figure 14:**
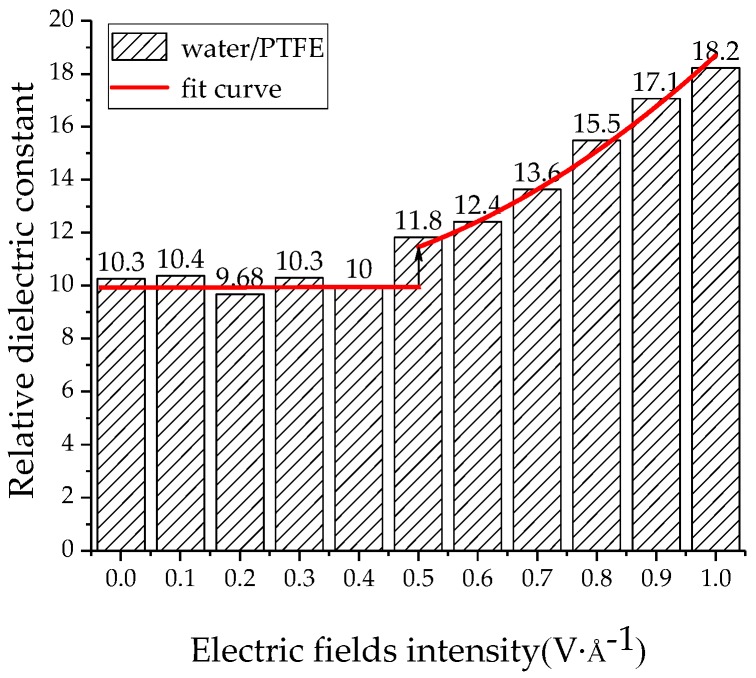
Variation of relative dielectric constant of the water/PTFE model with electric fields.

The relative dielectric constants of the PTFE model and the water/PTFE model at different electric field levels were calculated by applying 0~1 V/Å along the *z*-axis, and the increment step is 0.1 V/Å. As shown in Figure 13, when the electric field is 0~0.2 V/Å, the relative dielectric constant remains at about 2.2 for the PTFE model. When the electric field reaches 0.3 V/Å, the relative dielectric constant increases with increasing electric field intensity, and the variation law is a quadratic polynomial curve of *ε* = 0.33*E* + 3.65*E*^2^ + 2.2. As shown in Figure 14, the relative dielectric constant fluctuates around 10 for the 1% water/PTFE model when the electric field is in the range of 0~0.4 V/Å. When the electric field is greater than 0.4 V/Å, the relative dielectric constant increases with increasing the electric field intensity in a quadratic function, and the curve equation is *ε* = −3.55*E* + 12.01*E*^2^ + 10.23. Figure 13 and Figure 14 indicate that when the electric fields reach the critical field intensity, the electric field intensities have a great influence on the charge transfer and the molecular chain motion, and then the total dipole moment changes greatly, and the relative dielectric constant increases gradually with increasing electric field intensities. The critical field intensity of the PTFE model is 0.3 V/Å, and the critical field intensity of the water/PTFE model is 0.5 V/Å. When the electric field intensity is less than the critical field intensity, the electric fields intensities have little effect on the relative dielectric constant of the PTFE and water/PTFE models.

#### 3.4.2. Breakdown Strength

**Figure 15:**
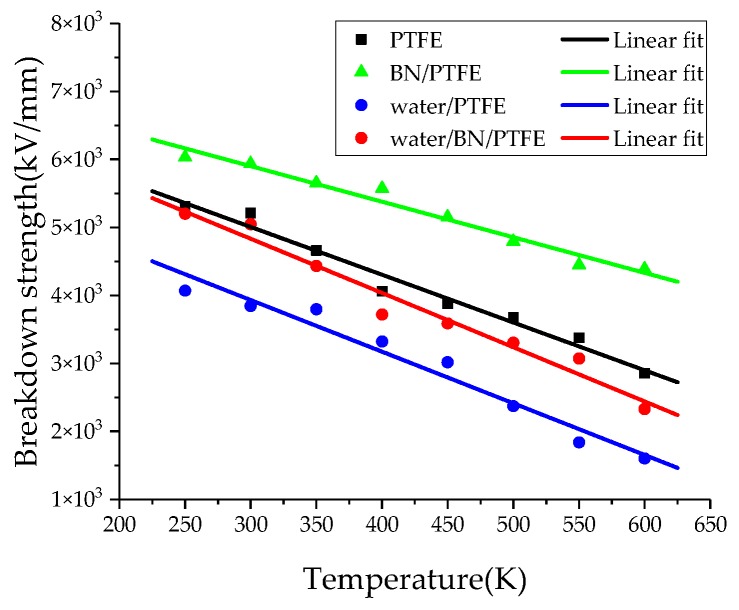
Breakdown strength versus temperature for four models.

Breakdown performance is one of the basic electrical properties of polymers that determines the ultimate ability of insulating materials to maintain insulation performance under electric fields. According to the electrical-mechanical breakdown theory proposed by Garton et al., the breakdown strength of PTFE can be approximately calculated by Young’s modulus *Y* according to the following equation [45,46]:

(14) $$E_{b}=0.6{\left(\frac{Y}{\varepsilon_{0}\varepsilon_{r}}\right)}^{\frac{1}{2}}$$

where *Y* is Young’s modulus, *ε*_0_ is the vacuum dielectric constant, and *ε_r_* is the relative dielectric constant. According to Equation (14), the dependence of breakdown strength on temperature for PTFE and its composite models can be obtained by MD simulation. The breakdown strength calculated by MD simulation is 5.21 × 10^3^ kV/mm, which is much larger than the experimental value of 42.4~90.1 kV/mm [47]. The difference between the experimental and simulation results is due to the different thickness of PTFE; the thickness of experimental sample is 75 μm while that of the simulation model is only 39.26 Å. Figure 15 shows that with the increase of temperature, the breakdown strength of PTFE and its composite models decreases. BN nanoparticles can significantly improve the breakdown strength of pure PTFE, and the more obvious effect of BN on PTFE breakdown strength is at higher temperature. However, the injection of water molecules can reduce the insulation strength of PTFE. The breakdown strength of the water/BN/PTFE model is higher than that of the water/PTFE model, which indicates that BN can reduce the effect of water on the breakdown strength of the PTFE model.

## 4. Conclusions

In this article, the glass transition temperature, mechanical properties, thermal conductivity, and dielectric properties of PTFE, BN/PTFE, water/PTFE, and water/BN/PTFE models are calculated and analyzed based on MD simulation. The following conclusions are drawn:

(1) The specific volume temperature method was used to determine *T*g. The incorporation of BN nanoparticles increases *T*g of PTFE by 25 K; the injection of water molecules reduces *T*g of PTFE by 22 K; and *T*g of water/BN/PTFE model is 27 K higher than that of water/PTFE model, which indicates that BN nanoparticles reduce the influence of water on the stability against degradation of PTFE materials.
(2) The mechanical properties of PTFE composite models at different temperatures were calculated by the static constant strain method. With the increase of temperature, Young’s modulus, shear modulus, and bulk modulus of the four models decrease gradually while Poisson’s ratio remains unchanged. The mechanical properties of the BN/PTFE model are obviously better than the PTFE model. The injection of water molecules reduces the mechanical properties of PTFE model by a large margin. However, BN nanoparticles have a certain inhibitory effect on the reduction of mechanical properties of PTFE materials after wet aging.
(3) The thermal conductivity of the PTFE model, BN/PTFE model, and grafted BN/PTFE model were calculated by RNEMD method. With the increase of temperature, the thermal conductivity of the three models increases almost linearly, and the incorporation of BN nanoparticles can improve the thermal conductivity of PTFE materials. Grafting the surface of BN nanoparticles can further improve the thermal conductivity of PTFE.
(4) The relative dielectric constant of the PTFE system can be calculated by using the total dipole moment of the PTFE system in the MD simulation process. With the increase of water content, the relative dielectric constants of the PTFE model and the BN/PTFE model increase gradually, and the latter increases less than the former, indicating that BN nanoparticles can inhibit the effect of wet aging on the relative dielectric constant of PTFE materials. Under electric fields, the dielectric constant of PTFE and its composite model firstly remains unchanged, and then they have an increasing trend of a quadratic polynomial curve when the electric field reaches critical field intensity. BN nanoparticles can effectively improve the breakdown strength of PTFE and reduce the influence of moisture on insulation strength.
